# Multiple effects of the bacterial DNA-binding protein SarA on the life cycle of *Staphylococcus aureus* phages

**DOI:** 10.1128/jb.00279-25

**Published:** 2025-10-16

**Authors:** Ronja Dobritz, Carina Rohmer, Elena Niepoth, Valentin Egle, Natalya Korn, Vittoria Bisanzio, Martin Saxtorph Bojer, Hanne Ingmer, Christiane Wolz

**Affiliations:** 1 Interfaculty Institute of Microbiology and Infection Medicine, University of Tübingen 9188, Tübingen, Germany; 2 Cluster of Excellence EXC 2124 “Controlling Microbes to Fight Infections”, University of Tübingen 9188, Tübingen, Germany; 3 Fraunhofer Institute for Interfacial Engineering and Biotechnology IGB 28453, Stuttgart, Germany; 4 Department of Veterinary and Animal Sciences, University of Copenhagen 4321, Copenhagen, Denmark; University of California San Francisco, San Francisco, California, USA

**Keywords:** *Staphylococcus aureus*, phage, SOS-response, DNA-binding proteins, SarA, Hlb

## Abstract

*Staphylococcus aureus* is a major opportunistic pathogen in humans and animals. More than 90% of human nasal *S. aureus* isolates carry Sa3int-phages that integrate into the bacterial *hlb* gene coding for a sphingomyelinase. Sa3int-phages encode highly human-specific virulence factors that enable *S. aureus* to adapt to the human host. Thus, balancing mechanisms are necessary for the phage-bacteria coexistence. However, the factors that coordinate these interactions have yet to be discovered. Here, we elucidate the impact of the DNA-binding protein SarA on the life cycle of two prototypic *S. aureus* phages, Sa3int Φ13 and Sa5int Φ11. SarA promotes the propagation of both phages, albeit via different mechanisms. SarA promotes Φ11 propagation by repressing the glycosyltransferase TarM, which affects the glycosylation pattern of the phage receptor, wall teichoic acid, thereby improving phage adsorption. SarA also dampens the DNA damage response as indicated by the downregulation of the *ci* and *mor* phage promoters and the *umuC* SOS target gene, as well as inhibition of Φ11 inducibility. For Φ13, however, SarA promotes phage replication rather than inhibiting phage induction. The replication-deficient phage Φ13K-*rep* was SarA-insensitive, and phage gene expression was unaltered in the *sarA* mutant. These results highlight SarA as a regulator of temperate phage propagation and support its role as a DNA structural protein that promotes phage replication.

**IMPORTANCE:**

The dynamic gain and loss of temperate phages is crucial for bacteria to adapt to specific niches. In *Staphylococcus aureus* Sa3int, phages are highly prevalent in human strains but are missing in most animal strains. The mechanisms that balance phage-bacteria coexistence are only partially understood. We demonstrate that the DNA-binding protein SarA is a key regulator of the phage life cycle. SarA protects bacteria from phage induction in response to DNA damage, yet it can also promote phage propagation by altering the phage receptor or interfering with phage replication. SarA likely functions not only as a transcriptional factor, but also as a bacterial chromosome structural component that controls the phage life cycle at different levels.

## INTRODUCTION

*Staphylococcus aureus* is a major opportunistic pathogen in humans and animals. This bacterium asymptomatically colonizes the nasal mucosa of healthy individuals but can cause life-threatening acute and chronic infections (1, 2). *S. aureus* has jumped between species many times, resulting in the dynamic gain and loss of host-specific adaptive genes, many of which are prophage encoded (2–5). Most prominent is the repeated loss of the temperate Sa3int phages after transfer of *S. aureus* from humans to different animals (6). In several instances, animal-adapted strains were transmitted back to humans, where they often reacquired Sa3int phages, emphasizing the importance of these temperate phages in human colonization (7). Up to 96% of human nasal *S. aureus* isolates carry Sa3int phages integrated into the *hlb* locus, which encodes the toxin ß-hemolysin (Hlb). While phage integration disrupts an important virulence factor of *S. aureus*, Sa3int phages carry genes that encode human-specific immune evasion factors and other potential virulence factors (6). The phage-encoded factors mediate the escape of *S. aureus* from human innate immunity (8). The observation that Hlb is always functional after phage excision and that this process also occurs during human infections, resulting in Hlb-positive sub-populations (9), indicates that under certain infectious conditions, Hlb is essential for bacterial survival. Recently, it was confirmed that phage excision enhances pathogenesis in mice (10). All temperate phages of *S. aureu*s are classified as siphophages (4). They can be discriminated into different groups based on their integrase gene allele (Sa-int groups) (11). The siphophage genomes are usually organized into six functional modules: lysogeny, DNA replication, packaging, head, tail, and lysis. The best-studied temperate phages from *S. aureus,* Sa5int phage Φ11 and the Sa3int phage Φ13, originate from the same host bacteria *S. aureus* NCTC 8325. Previously, we analyzed the life cycle of two Sa3int phages, Φ13 and ΦN315, in different phage-cured *S. aureus* strains. *S. aureus* strains could be classified into low (8325-4, SH1000, USA300c) and high (MW2c, Newman-c) transfer strains (12). Host–phage interactions probably account for the observed strain-specific differences in phage replication, assembly, and transfer frequency. The CI/Cro lysogenic/lytic switch in temperate phages is the best-studied regulatory switch to control phage mobilization and has been extensively characterized in the *Escherichia coli* phage λ. RecA-dependent cleavage of the CI repressor initiates phage induction and derepression of the lytic genes. Φ11 contains a λ-like CI/Cro switch region (13). In Φ13, the switch region is composed of a cleavable CI repressor and a Mor homolog (14). The small antirepressor Mor, first identified in lactococcal phages, functions as an anti-repressor of CI by protein–protein interaction (15, 16). The lytic state requires MOR-CI complex formation. Molecular circuits controlling the expression of late phage genes are only partially understood. Nevertheless, additional phage and host factors besides the “classical lytic switch” module are likely important for phage–host interaction. Active lysogeny or pseudolysogeny (13, 17) may be common for Sa3int phages and refers to the process whereby a prophage is temporarily excised from the chromosome without forming intact phage particles or the prophage is not integrated following infection. This allows bacteria to simultaneously express phage-encoded virulence genes, as well as the gene that is usually inactivated by prophage integration, e.g., the *hlb* gene (9).

Recently described xenogenic silencing factors (XS) may contribute to balancing bacteria-phage co-existence (18), particularly favoring and maintaining the lysogenic state. The XS factors discovered so far are small, nucleoid-associated proteins that recognize and bind AT-rich DNA stretches. Of note, none of the described factors are present in *S. aureus*. We speculated that SarA family proteins might fulfill an XS-like function. SarA recognizes and binds AT-rich DNA motifs and is usually seen as a transcriptional factor controlling gene expression (19, 20). However, SarA seems to bind the chromosome more frequently than one might expect for a bona fide transcription factor. SarA is present at intracellular concentrations far exceeding any classical transcription factor, and its concentration remains unchanged during different growth phases (21). SarA was therefore suggested to be a histone-like protein that may alter DNA topology (21, 22).

Here, we analyzed the role of SarA in the life cycle of two prototypic siphophages, Φ11 and Φ13. Our data indicate that SarA promotes propagation of both phages, albeit in different ways. By inhibiting the expression of glycosyltransferase TarM, SarA alters the glycosylation pattern of the phage receptor wall teichoic acid (WTA) and promotes Φ11 adsorption. In contrast, the positive impact of SarA on Φ13 replication can be attributed to the promotion of a step between prophage excision and viral particle assembly, which most likely requires the function of SarA as a DNA structural protein rather than a transcription factor.

## RESULTS

### SarA promotes propagation of siphophages Φ11 and Φ13

**Fig 1:**
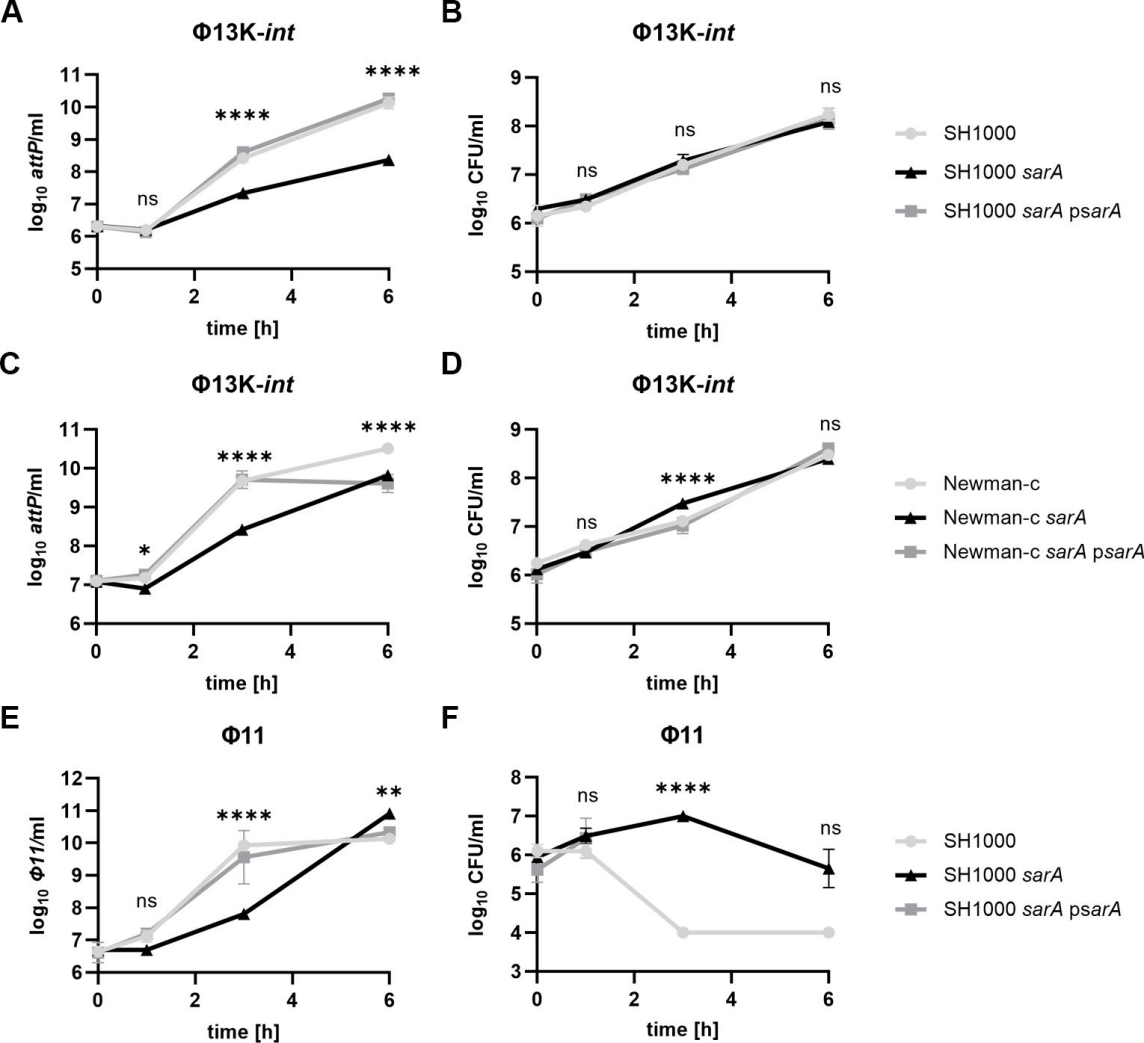
SarA promotes propagation of Φ11 and Φ13. Phage replication and bacterial growth in wild type, *sarA* (*sarA::ermC* deletion), and *sarA* p*sarA* (*sarA* complementation) following phage infection. Phage-free strains were grown to an OD_600_ of 0.5, then 1 × 10^6^ bacteria per milliliter were infected with integrase-deficient Φ13K-*int* (**A, B, C, D**) or Φ11 (**E, F**) at MOI 1 and incubated for up to 6 h. Phage replication was analyzed by enumerating free phage genomes in the supernatant by quantitative PCR (qPCR) (copies of *attP*) (**A, C, E**), and bacterial growth was monitored by colony-forming unit (CFU) determination (**B, D, F**). Data shown are mean ± SD (*n* = 3). Statistical significance was determined by two-way ANOVA tests on log_10_-transformed data for multiple comparisons for each time point (Tukey’s multiple comparison test). Statistical results for wild type vs *sarA* comparison are shown (****P value < 0.0001, **P value < 0.01, ns > 0.05).

We analyzed SarA-dependent phage propagation using two phage-cured *S. aureus* strains (SH1000 and Newman-c). *SarA* mutants were generated, and mutants were complemented by integration of the *sarA* expressing plasmid into the *geh* locus. We first analyzed the infection dynamics of phage Φ13K-*int* (23), a kanamycin-resistant derivative of Φ13, the native phage of the SH1000 ancestor NCTC 8325 (12). Unlike Φ13K, Φ13K-*int* lacks the integrase and thus produces clear plaques because it cannot enter the lysogenic life cycle. After infection at a multiplicity of infection (MOI) of 1, phage replication was significantly lower in the *sarA* mutants compared to wild-type strains (SH1000, Newman-c) or complemented mutants (Fig. 1A and C). Despite phage propagation, little or no concomitant bacterial lysis was detectable after infection, and bacterial numbers continued to increase up to 6 h post-infection (Fig. 1B and D). This is probably due to the phage infecting only a sub-population of bacteria or to phage replication/assembly being somehow inhibited in most of the bacterial population. However, in strain Newman, a slight but significant SarA-dependent inhibition of bacterial replication was detectable in the wild-type and complemented strains 3 h post-infection. These data indicate that host-derived factors modulate Φ13K-*int* propagation.

We next analyzed the infection dynamics of Φ11, a phage from the Sa5int group. Like Φ13, this phage is also derived from the SH1000 parental strain NCTC 8325. Following phage infection, significantly fewer phage copies were detectable in the *sarA* mutant 3 h post-infection (Fig. 1E). However, in contrast to Φ13K-*int,* infection resulted in bacterial lysis of the wild-type and complemented strain (Fig. 1F). In the *sarA* mutant, bacterial lysis was partial and detectable only after >3 h of infection. Thus, SarA promotes phage propagation of Φ13K-*int* and Φ11, despite phage-specific differences in host cell lysis.

### SarA supports adsorption of Φ11 but not Φ13K

**Fig 2:**
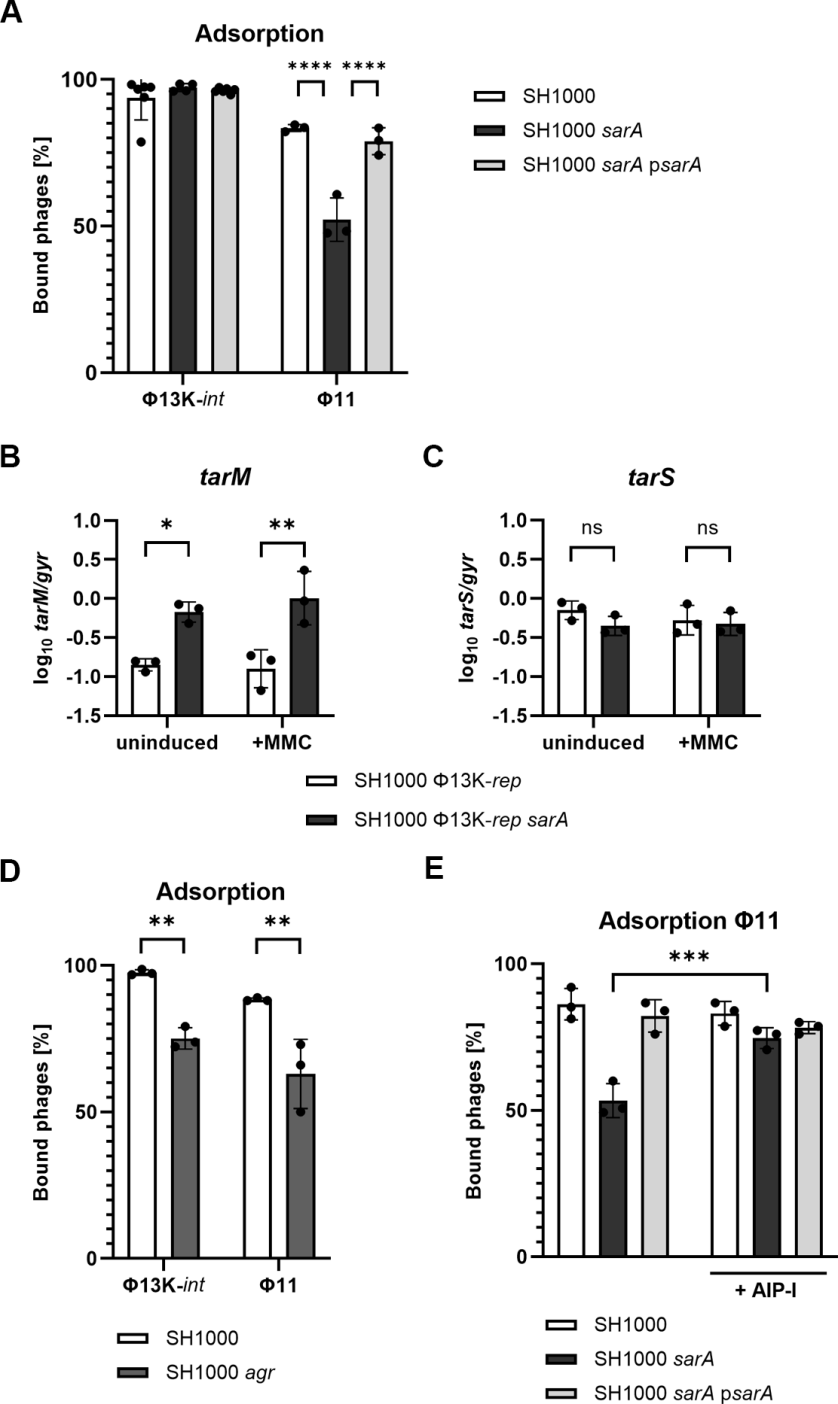
*sarA* deletion results in reduced binding of Φ11 and altered expression of WTA glycosyl transferase *tarM*. (**A**) Phage adsorption of Φ13K-*int* and Φ11 to SH1000 (wild type), SH1000 *sarA* (*sarA::ermC* deletion), and SH1000 *sarA* p*sarA* (*sarA* complementation). Phage-free strains were grown to an OD_600_ of 1, and 1 × 10^8^ bacteria per milliliter were incubated for 10 min with phages at MOI 0.01. Unbound phages were enumerated by qPCR on the phage genome and compared to media control to calculate the number of bound phages. (**B, C**) Gene expression analysis of *tarM* (α-1,4 GlcNAc) and *tarS* (β-1,4 GlcNAc) under uninduced and induced (+MMC) conditions. Single-lysogenic SH1000 and SH1000 *sarA* (*sarA::ermC* deletion), containing the replication-deficient Φ13K-*rep* mutant, were grown to exponential growth phase, followed by prophage induction with subinhibitory mitomycin C (MMC), and incubation for 60 min. RNA was isolated, *tarM* and *tarS* transcripts were quantified by quantitative reverse transcription polymerase chain reaction (qRT-PCR), and normalized to *gyr* expression. (**D**) Phage adsorption of Φ13K-*int* and Φ11 to SH1000 (wild type) and SH1000 *agr* (*agr::tetM* deletion). (**E**) Phage adsorption of Φ11 to SH1000, SH1000 *sarA,* and SH1000 *sarA* p*sarA* in the presence of AgrA, inducing peptide, AIP-I. A total of 100 nM AIP-I was added to the precultures. Data shown are mean ± SD (*n* = 3). Statistical significance was determined by a two-way ANOVA test (***P value < 0.001, **P value < 0.01, *P value < 0.05, ns > 0.05).

We next analyzed the impact of SarA on phage adsorption, a prerequisite for phage propagation. After 10 min of phage adsorption, the number of unbound phages was determined by quantitative PCR (qPCR) on phage genomes (Fig. 2A). While phage adsorption of Φ13K-*int* was not altered in the *sarA* mutant, adsorption of Φ11 to the *sarA* mutant was significantly reduced. WTA serves as a receptor for all *S. aureus* phages, although differences exist between phages with respect to the specific WTA moiety bound (24). Φ11 adsorption is dependent on WTA glycosylation, whereas Φ13 binds to the RboP-WTA backbone, irrespective of its glycosylation pattern. Glycosylation is catalyzed by the glycosyltransferases TarM and/or TarS. We speculated that SarA might impact the expression of *tarS* and/or *tarM* and thereby modulate Φ11 adsorption. Expression of *tarM* was found to be significantly increased in the *sarA* mutant, whereas *tarS* expression was SarA-independent (Fig. 2B and C). Complementation of the *sarA* mutant restored *tarM* expression to the level of the wild type (Fig. S1). Since SarA controls the activity of the quorum-sensing system Agr (25) and Agr inhibits *tarM* expression (26), we speculated that SarA's effect on Φ11 adsorption is due to Agr-dependent dysregulation of *tarM*. Indeed, *sarA* and an isogenic *agr* mutant were similarly diminished in Φ11 adsorption (compare Fig. 2A and D). Furthermore, incubating the *sarA* mutant with the autoinducing peptide AIP-I counteracted the SarA-mediated inhibition of phage adsorption (Fig. 2E).

Interestingly, Φ13 adsorption was also found to be *agr* dependent, although *sarA* deletion did not significantly alter phage binding (compare Fig. 2A and D).

The data indicate that SarA-dependent promotion of Φ11 propagation is due to enhanced phage adsorption, likely due to Agr-mediated inhibition of *tarM*. However, adsorption of Φ13 is glycosylation-insensitive and SarA-independent. Thus, SarA likely impacts Φ13 life cycle via mechanism(s) unrelated to phage adsorption or other Agr-mediated effects.

### SarA promotes replication of Φ13K but not Φ11

**Fig 3:**
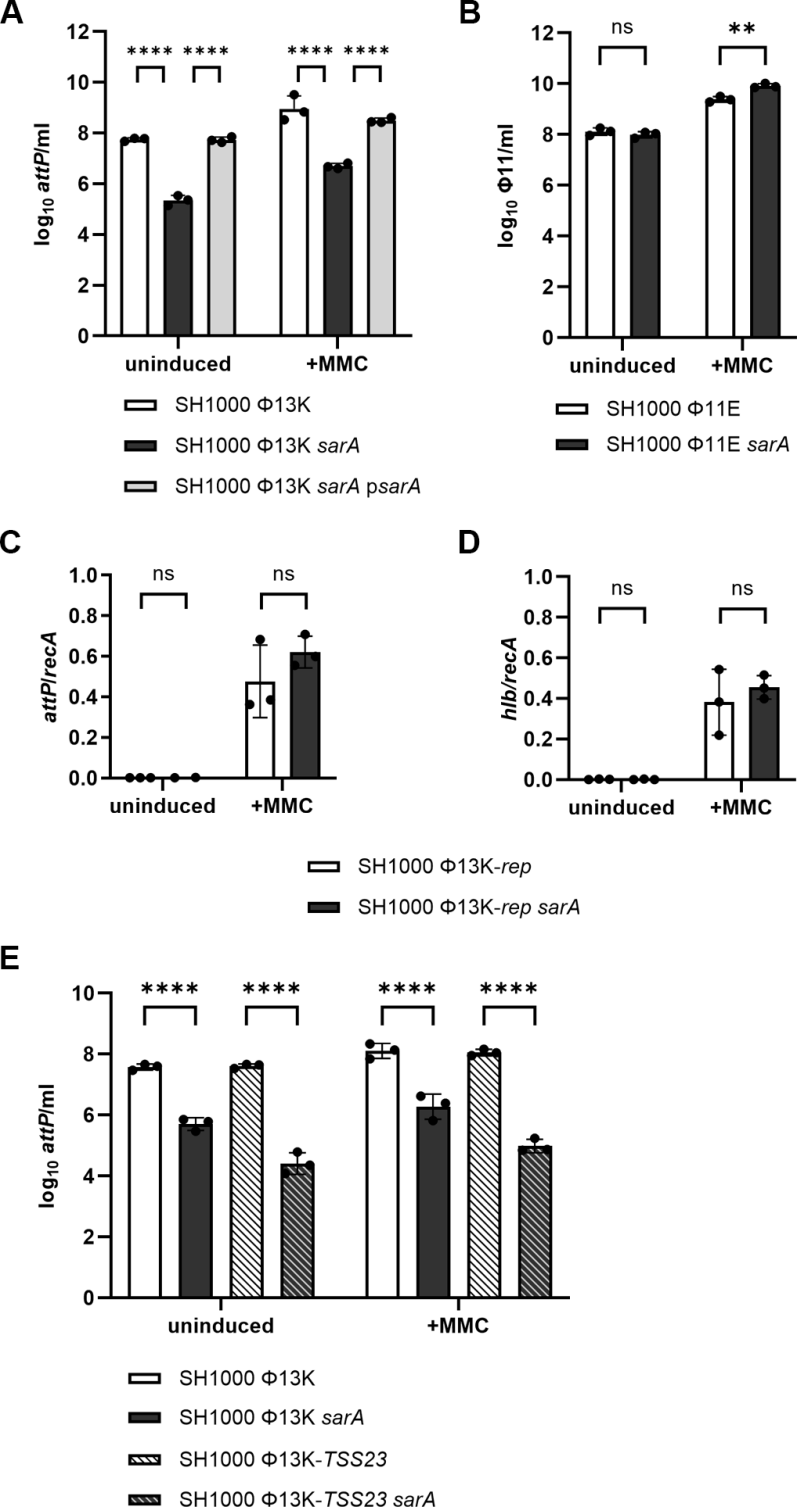
SarA supports Φ13K phage replication. Phage replication in single-lysogenic SH1000 (wild type), SH1000 *sarA* (*sarA::ermC* deletion), and SH1000 *sarA* p*sarA* (*sarA* complementation) under uninduced and induced (+MMC) conditions. (**A**) Single-lysogenic Φ13K strains were induced with subinhibitory MMC in exponential growth phase and incubated for 60 min. Phage numbers were determined by qPCR (*attP*). (**B**) Single-lysogenic Φ11 strains were grown to the exponential growth phase, induced with subinhibitory MMC, and incubated for 60 min. Phage numbers were determined by qPCR on the phage genome. (**C, D**) Intracellular phage excision and replication. Replication-deficient (-*rep*) Φ13K-*rep* single-lysogenic strains were induced with subinhibitory concentrations of MMC and incubated for 60 min. Intracellular phage replication was enumerated as the ratio of excised, circularized phage genomes (*attP*) per copy of bacterial chromosome (*recA*). Phage excision was quantified by qPCR on reconstituted *hlb* genes per bacterial chromosome (*recA*). (**E**) Single-lysogenic Φ13K or Φ13K-*TSS23* (non-infectious phage mutant) strains were grown to exponential growth phase, induced with subinhibitory MMC, and incubated for 60 min. Phage numbers were determined by qPCR on the phage genome. Data shown are mean ± SD (*n* = 3). Statistical significance was determined by two-way ANOVA tests on log_10_-transformed data (****P value < 0.0001, **P value < 0.01, ns > 0.05).

To further analyze the impact of SarA on the phage life cycle, *sarA* mutants were created in single-lysogens of strain SH1000. We first analyzed the induction of Φ13K with and without the inducing agent mitomycin C (MMC). The extracellular genome copies of released Φ13K from culture supernatants were quantified by attachment site (*attP*/mL) qPCR. SarA deletion significantly reduced the number of released Φ13K copies (239-fold decrease under uninduced and 278-fold under induced conditions), a phenotype that was fully reverted to wild type in the complemented strain (Fig. 3A). Interestingly, no difference in replication of Φ13K between wild-type and *agr* mutant was observed, supporting the hypothesis that the effect of SarA on phage replication is Agr independent (Fig. S2).

We next analyzed phage excision of Φ11E (erythromycin-resistant derivative of Φ11) lysogen generated in strain SH1000 and its *sarA* mutant. No significant difference in phage excision/replication between wild-type and *sarA* mutant was detectable under uninduced conditions (Fig. 3B). After MMC induction, Φ11 copies were increased in the *sarA* mutant in comparison to the wild type, which sharply contrasts the results obtained for Φ13K (Fig. 3A and B). The data underline that SarA differentially impacts the life cycle of the analyzed phages.

To dissect whether SarA impacts phage excision, replication, or assembly of Φ13, we quantified phage excision in replication-deficient phage lysogens (Φ13K-*rep*) (12). Φ13K-rep can still be excised from the chromosome but is unable to replicate or produce intact phage particles. The number of excised phage copies was comparable to the number of restored *hlb* copies in the bacterial population (Fig. 3C and D). No significant SarA-dependent differences were found in phage excision or *hlb* reconstitution. Thus, processes following phage excision are likely to be controlled by SarA.

We next analyzed a phage mutant (-*TSS23*) that can excise and replicate but cannot assemble due to mutations in the main promoter (p23) controlling the expression of structural genes. Consequently, induction of this phage does not result in plaque formation. A significant difference in the replication of phage Φ13K-*TSS23* was observed between the wild-type and *sarA* mutant, comparable to the difference detected for the wild-type Φ13K (Fig. 3E). These results indicate that SarA affects a step between excision of the prophage and assembly of intact phage particles, which accounts for the significant reduction in the number of wild-type Φ13K phage copies released in the *sarA* mutant (Fig. 3A).

### SarA does not impact supercoiling or protease-dependent phage decay

Overexpression of proteases is a main feature of *sarA* mutant strains (27), and *S. aureus* phages were shown to be protease sensitive (28). We confirmed that the *sarA* mutant has significantly higher proteolytic activity than the wild type (Fig. S3). We speculated that phages might be readily degraded in the supernatant of *sarA* mutant strains, which would explain the decrease in phage infectivity (Fig. 3A). However, Φ13 particles after incubation in culture supernatants from wild-type or the *sarA* mutant were equally stable (Fig. S3). Next, we speculated that SarA might function as a structural DNA-binding protein that modifies supercoiling and thereby may impact phage gene transcription or replication. However, supercoiling of a small plasmid that acts as a monitor for the global level of supercoiling in the cell was not found to differ between wild-type and *sarA* mutant (Fig. S4).

### SarA inhibits promoter activities of the phage switch region and the SOS gene *umu*C

**Fig 4:**
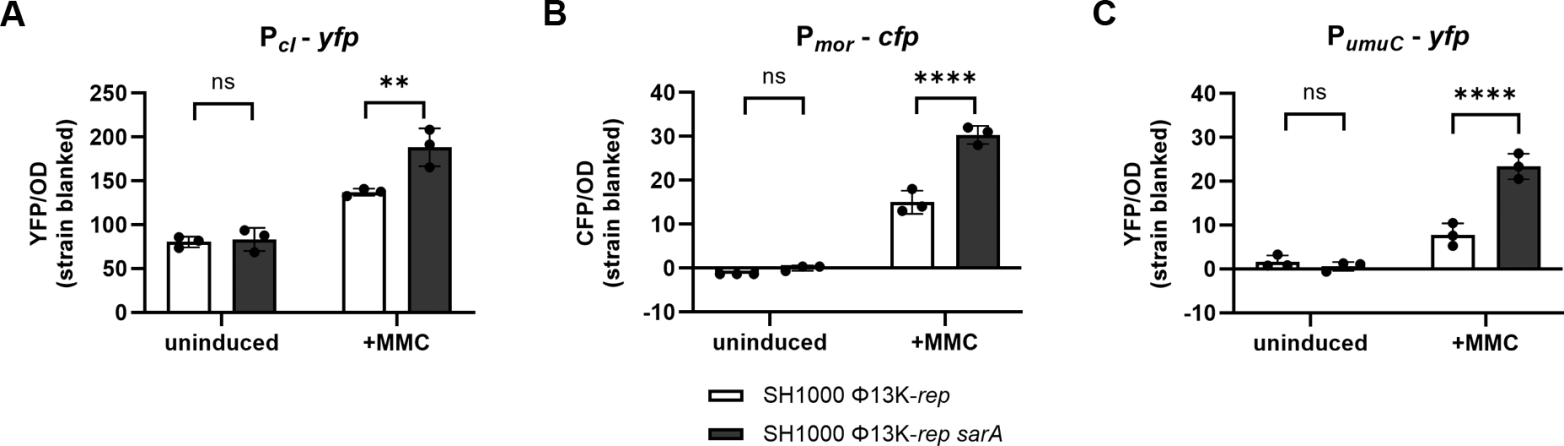
DNA-damage response activation in the *sarA* mutant background. Promoter activity of P*_cI_*, P*_mor_*, and P*_umuC_* in single-lysogenic SH1000 Φ13K and SH1000 Φ13K *sarA* (*sarA::ermC* deletion) under uninduced and induced (+MMC) conditions. (**A–C**) Reporter plasmid (P*_cI_*: pCG748, P*_mor_*: pCG789, P*_umuC_*: pCG762) containing strains were grown to the exponential growth phase, induced with subinhibitory MMC, and incubated for 60 min. Bacterial cultures were harvested to an OD_600_ of 2 and resuspended in phosphate-buffered saline (PBS). Optical density and fluorescence were measured. Arbitrary units of fluorescence (yellow: YFP, blue: CFP) are shown, normalized to OD_600_; strain-specific background fluorescence was subtracted. Data shown are mean ± SD (*n* = 3). Statistical significance was determined by two-way ANOVA tests (****P value < 0.0001, **P value < 0.01, ns > 0.05).

To clarify how SarA promotes Φ13 replication, we analyzed whether phage gene expression is affected in a *sarA*-dependent manner. First, we used promoter fusion constructs of the lytic switch region of Φ13. *CI* and *mor* promoters containing CI binding sites were fused to *yfp* and *cfp,* respectively (P*_cI_-yfp* and P*_mor_-cfp*). Promoter activities were analyzed in Φ13K-*rep* lysogens of SH1000 and its *sarA* mutant. Under uninduced conditions, promoter activities were low, and no significant difference between wild-type and *sarA* mutants was observed. However, after MMC treatment, the activities of both phage promoters were significantly increased in the *sarA* mutants (Fig. 4A and B). This indicates that RecA-dependent CI cleavage or RecA activity might be repressed by SarA. If RecA activity is hampered, the LexA-dependent SOS response should also be altered in the *sarA* mutant. Accordingly, the activity of the *umuC* promoter*,* a prototypic LexA target gene in *S. aureus,* was also significantly increased in the *sarA* mutant after MMC induction, indicating enhanced SOS-response activation (Fig. 4C). The increased *umuC* expression in the *sarA* mutant after MMC treatment was confirmed by RT-qPCR (Fig. S5).

The results indicate that SarA dampens the SOS response and the lytic phage life cycle. This would be consistent with an increase in phage mobilization in *sarA* mutants as observed for Φ11 (Fig. 3B). However, the finding is counterintuitive to the observation that replication of Φ13 is decreased in the *sarA* mutant (Fig. 3A). Only for the replication-deficient phage Φ13K-*rep,* a slight increase in phage induction was detectable in the *sarA* mutant (Fig. 3C).

### SarA does not control gene expression of Φ13K

**Fig 5:**
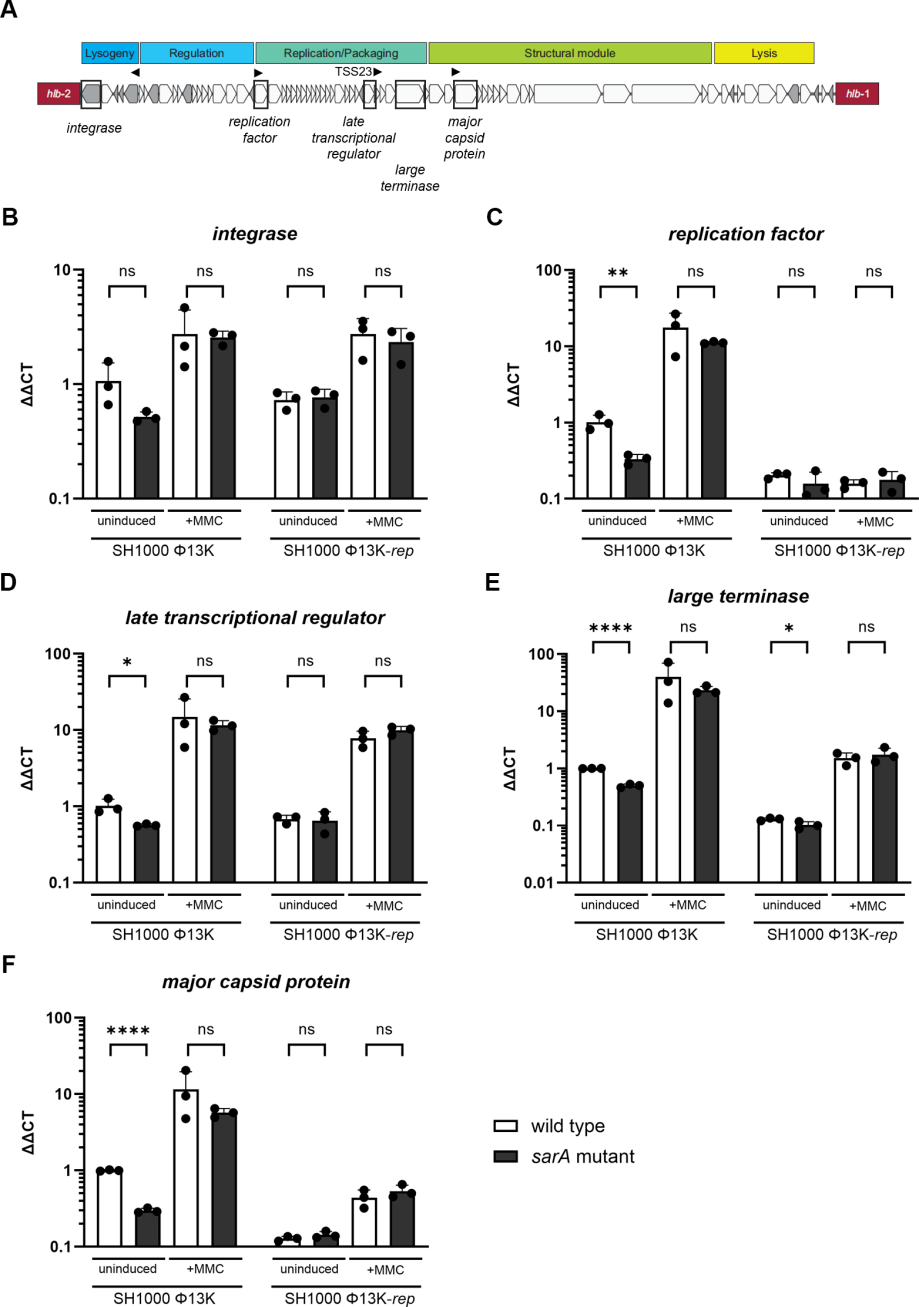
SarA does not control phage gene expression of Φ13K. Gene expression analysis of phage genes under uninduced and induced (+MMC) conditions. (**A**) Schematic depiction of gene organization in the Φ13K genome. Transcriptional start sites are displayed as arrowheads. (**B–F**) Single-lysogenic SH1000 and SH1000 *sarA* (*sarA::ermC* deletion) containing wild-type or replication-deficient Φ13K (Φ13K-*rep*) were grown to exponential growth phase, induced with subinhibitory MMC, and incubated for 60 min. RNA was isolated, and *integrase*, *replication factor*, *late transcriptional regulator*, *large terminase,* and *major capsid protein* transcripts were quantified by RT-qPCR and normalized to *gyrB* expression by the ΔΔCt method. Data shown are mean ± SD (*n* = 3). Statistical significance was determined by unpaired *t*-tests comparing wild-type and *sarA* mutant (****P value < 0.0001, *P value < 0.05, ns > 0.05).

To account for the SarA-dependent promotion of Φ13K replication, we speculated that SarA might control the expression of phage genes. Therefore, we analyzed phage gene expression by quantitative reverse transcription polymerase chain reaction (RT-qPCR) under induced and uninduced conditions. We selected a set of genes from different modules of Φ13K and initiated by different transcriptional start sites (12) (Fig. 5A). Expression of genes was significantly lower in the *sarA* mutant. However, this might be simply due to reduced phage genome copy numbers, as phage replication is decreased in the *sarA* mutant. Thus, we also analyzed gene expression in the replication-deficient Φ13K-*rep* lysogens. In these lysogens, no SarA-dependent effects on phage gene expression were detectable (Fig. 5B through 5F). Thus, SarA does not impact phage gene expression. Instead, it is more likely to impact Φ13K phage propagation by interfering with DNA-dependent processes, such as initiation or elongation of replication.

## DISCUSSION

**Fig 6:**
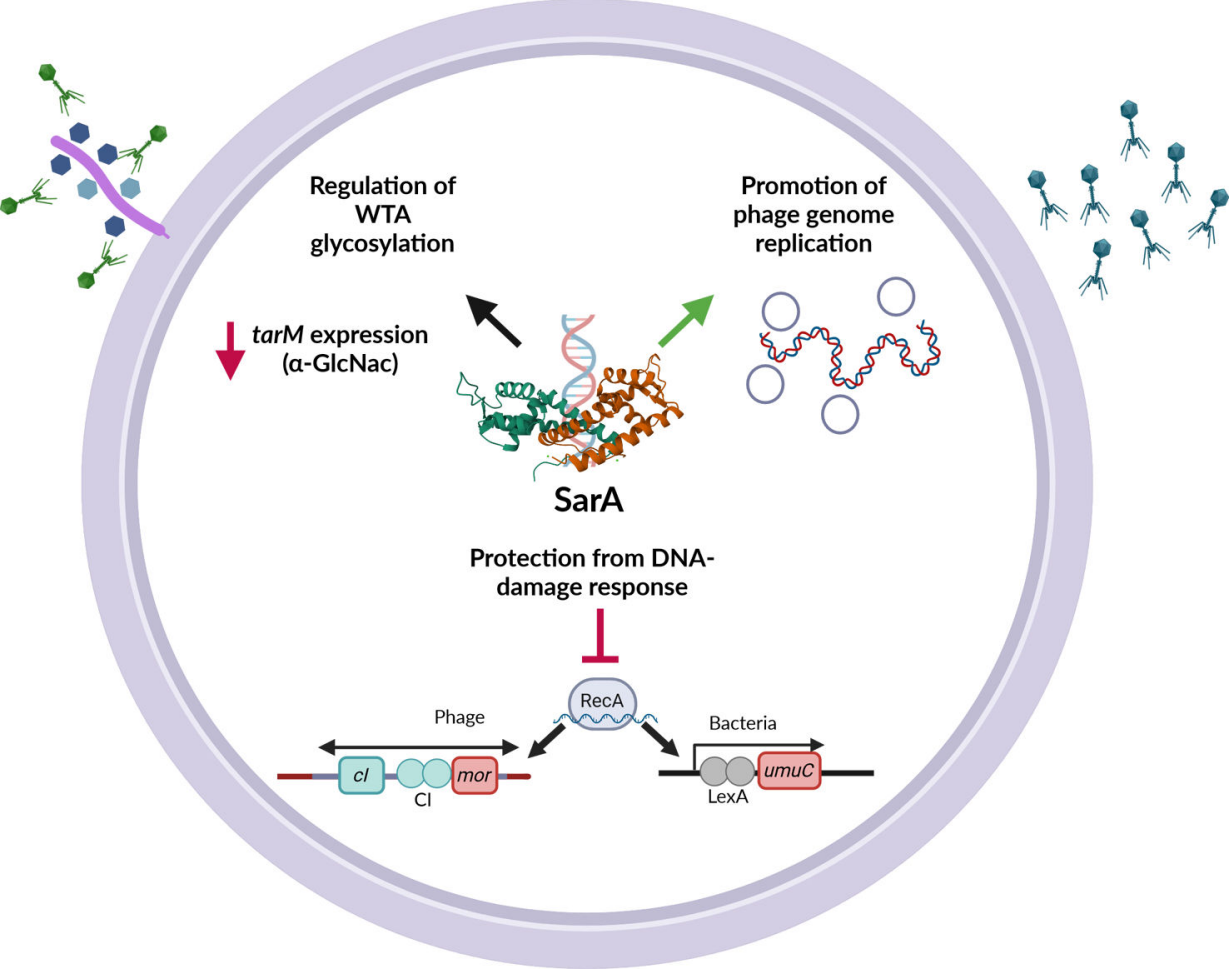
Multiple effects of the bacterial DNA-binding protein SarA on the life cycle of *S. aureus* phages. SarA promotes phage infection and replication on various levels. Repression of glycosyltransferase TarM affects the glycosylation pattern of WTA and thereby facilitates binding of Φ11. SarA protects the phages and bacteria from DNA-damage response leading to a lower genetic switch- and SOS-response activation. Additionally, the phage replication of Φ13 is promoted by SarA, resulting in higher numbers of phages. Crystal structure of SarA downloaded from RCSB Protein Data Bank (Entry ID:2FRH). Image created with BioRender.com.

Temperate phages, such as Sa3int phages, fulfill beneficial functions for their bacterial hosts, but they also pose a threat to the host bacterium once mobilized. Thus, balancing mechanisms are necessary for the co-existence of phages with their hosts, as well as for phage-dependent bacterial adaptation to specific niches. Such mechanisms are not well understood, and putative bacterial factors that coordinate these interactions have not yet been described for highly prevalent Sa3int phages. We show that SarA promotes the propagation of two prototypic phages of *S. aureus*, Φ11 and Φ13, although via different mechanisms (Fig. 6).

### SarA promotes Φ11 phage adsorption through *tarM* inhibition

Phage adsorption depends on the tight interaction of phage receptor-binding proteins (RBPs) and WTA. RBPs of *S. aureus* phages can be grouped into eight distinct clusters (24). Φ11 contains two RBPs and requires WTA glycosylation for binding. Binding of Φ11 was significantly decreased in the *sarA* mutant. We propose that the SarA-mediated promotion of Φ11 binding is due to SarA/Agr-dependent inhibition of *tarM* expression. This is supported by the following findings. *SarA* and a*gr* mutants show similar inhibition of Φ11 binding, and the *sarA* mutant could be complemented by the addition of AIP-I, indicating that SarA may at least in part function via Agr activation (25). Recently, it was shown that Agr quorum-sensing induction reduces TarM-mediated α-GlcNAc glycosylation of WTA and that α-GlcNAc glycosylated WTA can hinder infection of some phages (26). In a transposon screen using glycosylation-specific antibodies, *sarA* and *agr* mutants were identified as exhibiting higher levels of α-GlcNAc glycosylation (29). Reduced TarM-dependent α-GlcNAc glycosylation leads to glycoswitching, favoring β-GlcNAc glycosylation via TarS. Moreover, the inhibition of *tarM* expression via the two-component system *arlRS* also promotes the infection of glycosylation-dependent phages. Our data indicate that the *sarA* mutant exhibits a phenotype similar to the *arlRS* mutant, resulting in the expression of *tarM*, which in turn hinders Φ11 adsorption. Additionally, for successful adsorption, the efficient cleavage of peptidoglycan (PG) might play a role in phage genome injection and subsequent replication. However, there is little evidence for the role of hydrolases in *S. aureus* phage infection. Indeed, the putative hydrolase of Φ11 was shown to be dispensable for successful phage infection (30). For Φ13, no gene similar to the already identified PG hydrolases (31) could be identified, thus a potential effect of SarA on cell wall molecules through PG cleavage is not likely. In contrast to the RBP from Φ11, the RBP of Φ13 was shown to bind WTA independent of its glycosylation status (24). This finding is consistent with our results that phage adsorption is SarA- and glycosylation-independent. Surprisingly, mutation of *agr* but not *sarA* resulted in decreased adsorption of Φ13. Agr was shown to increase WTA surface expression through activation of *tarH* and the chain length through *tarK* (32). One can speculate that such Agr effects on WTA are promoting Φ13K-*int* phage adsorption and that Agr regulation of *tarH* and/or *tarK* only requires basal Agr activity as present in the *sarA* mutant.

### SarA dampens the SOS response and phage repressor gene expression

In the search for additional SarA-dependent factors controlling phage propagation, we found that SarA dampens the expression of phage repressor gene *cI* and its antirepressor *mor,* as well as the LexA target gene *umuC*. CI and LexA are both repressors that are activated through autocleavage upon DNA damage-induced RecA activation. SarA effects were only detectable after MMC treatment, indicating that SarA restricts either MMC-induced DNA damage or the consequent RecA activation. A DNA-safeguarding role for SarA is supported by the finding that SarA protects *S. aureus* from the DNA-damaging antibiotic ciprofloxacin (33). Consistent with the hypothesis that SarA antagonizes DNA damage, we found an increase in intracellular Φ11 copy numbers after MMC induction in *sarA* mutants (Fig. 3B). A slight increase in phage induction was also observed for the replication-deficient Φ13K-*rep* phage (Fig. 3C). However, for the wild-type Φ13K, reduced phage copy numbers were detected in the *sarA* mutants, indicating that SarA-dependent promotion of replication of this phage is controlled by other, more dominant mechanisms.

### SarA promotes phage replication of Φ13K

The main effect of SarA on the Φ13 life cycle is likely due to its promotion of phage replication. In *sarA* mutants, phage propagation after infection is delayed, and phage release from lysogenic bacteria is inhibited. This could not be linked to differences in phage adsorption, susceptibility to proteases, phage assembly, phage excision, or differences in RecA activity. For the Φ13K-*rep* mutant, no SarA-dependent effects on phage induction were detectable, and we could not detect significant differences in phage mRNA levels for the non-replicative phage. Thus, SarA's impact on phage replication is likely not caused by its function as a classical transcriptional factor or as a modulator of transcript stability as previously proposed (19, 34, 35). We conclude that SarA is more likely to increase phage copy numbers by directly interfering with phage replication. Secondary effects on SarA-dependent changes in physiology might have contributed to better replication conditions in SarA-expressing strains. However, there is little evidence that SarA impacts bacterial growth as wild-type and *sarA* mutants do not differ in their growth rate (e.g., see Fig. 1B, D, and F). Moreover, we would expect this to be a more general feature also affecting replication of Φ11 after induction of prophages, for which we found no evidence.

SarA was suggested to be a histone-like protein that may alter DNA topology (21, 22). This was supported by the observation that SarA is present at intracellular concentrations far exceeding any classical transcription factor, and its concentration remains essentially unchanged during different growth phases (21). One may speculate that SarA binding to extrachromosomal phage DNA somehow differs from binding to chromosomal DNA. SarA binding properties are also dependent on the phage analyzed since we have no evidence that Φ11 replication is influenced by SarA. Interestingly, ChIP-Seq indicates that SarA tends to cluster in certain regions of the chromosome (20). A pathogenic island appeared as a privileged SarA binding zone, supporting that SarA binding is dependent on certain features of DNA topology. However, SarA does not seem to alter DNA supercoiling. It was previously shown that when expressed in *E. coli,* SarA specifically recognizes the *att* site of phage λ (21). Thus, the detailed mechanisms by which the interaction between SarA and the phage impacts phage replication remain to be elucidated. SarA may specifically interfere with the origin of replication, thereby promoting replication initiation and/or elongation. However, the ori for Φ13 replication has yet to be defined, hampering a closer molecular follow-up of this hypothesis. We initiated this study with the hypothesis that SarA might function as an XS factor (18). Currently known XS proteins are small proteins that fall into four classes (H-NS, MvaT, Rok, and Lsr2). They have been shown to follow a common mode of action by binding to AT-rich DNA, forming an oligomeric nucleoprotein complex, and are likely targeted by post-translational modification enzymes. These are all features shared with SarA, which is modified via cysteine phosphorylation (36). However, the known XS factors are described to promote the lysogenic state of phages. SarA instead promotes phage replication, thus functioning as a phage replication factor rather than a silencing factor.

### Conclusion

The life cycle of temperate phages must be tightly controlled. Here, we got first insights into the specific host–phage interaction for two prototypic temperate *S. aureus* phages. The DNA-binding protein SarA interferes with phage propagation at several levels. SarA promotes Φ11 propagation via altering WTA glycosylation. However, for Φ13, SarA mainly promotes phage replication. This indicates that SarA is not only a transcriptional factor but also functions as a DNA structural protein. Such a function could also explain the DNA protective role of SarA. Future studies will help clarify the exact molecular mechanisms underlying the observed effects of SarA on temperate phage replication. SarA is an important modulator of the phage life cycle, which, on the one side, protects the bacteria from phage induction upon DNA damage but can promote phage propagation via alteration of the phage receptor or interference with phage replication.

## MATERIALS AND METHODS

### Growth conditions

Strains and phages used in this work are listed in Tables S1 and S2. If not stated differently, strains were grown in tryptic soy broth (TSB) (Oxoid) at 37°C and 200 rpm. For strains carrying resistance genes, antibiotics (erythromycin [erm] 10 µg mL^−1^, tetracycline [tet] 3 µg mL^−1^, kanamycin [kan] 50 µg mL^−1^, chloramphenicol [cm] 10 µg mL^−1^) were used in precultures and for selection.

### Generation of strains and mutants

Oligonucleotides used for cloning and generation of strains are listed in Table S3.

#### Generation of sarA and agr mutants

In order to generate *sarA* and *agr* mutants, phage lysates from ALC1342 and RN6911 were generated, and recipient strains were transduced. Correct mutations were confirmed by PCR spanning the mutation site.

#### Generation of sarA-complemented strains

The *sarA* locus (*sarAP1-P3*, *sarA*) was cloned into the integration plasmid pLL39 by amplification with primers pCG921gibfor/rev and following Gibson Assembly into the BamHI-digested backbone plasmid. The resulting plasmid pCG921 was integrated into the *geh* site of CYL316 by electroporation (37, 38). Subsequently, the *sarA* plasmid was introduced into *sarA* mutants by Φ11 transduction.

#### Generation of single-lysogenic SH1000 Φ11E

Phage lysate of Φ11 containing an erm cassette was generated by induction of single-lysogenic 8325-4 Φ11E. Bacteria in the early exponential phase were incubated with phages at an MOI of 1 for 4 h. Serial dilutions of the culture in phosphate-buffered saline (PBS) were spotted onto agar plates containing erm (10 µg mL^-1^). Single-lysogens were sub-cultivated for 5 days, and stable phage integration was confirmed by PCR.

#### Promoter fusion constructs for fluorescence measurements

Promoter regions of genes of interest (P_gene_) were cloned in front of a strong ribosomal binding site (RBS) and genes encoding for fluorescent proteins (gpVenus, gpCerulean) by Gibson Assembly. For P_cI_-*yfp* (pCG748), 130 bps upstream of the putative RBS were amplified with primer pcIyfpgibfor/pcIyfpgibrev and cloned into reporter plasmid pCG725 (P_cap_-*yfp*) digested with SalI and SphI to replace P*_cap_* (32). For P_mor_-*cfp* (pCG789), the backbone reporter plasmid pCG733 (P*_cap_-cfp*) was digested with SphI and EcoRI, 133 bp upstream of the RBS of *mor* were amplified by PCR with primer pCG789gibfor/pCG789gibrev and cloned into the backbone vector. For P*_umuC_-yfp* (pCG762), the promoter region, consisting of 130 bp upstream of the RBS of *umuC*, was amplified using primers pumuCYFPgibfor/pumuCYFPgibrev. The resulting plasmids were verified by PCR and sequencing and introduced into RN4220 for transduction or electroporated directly into the final strains.

#### Non-infectious phage mutant (Φ13K-TSS23)

Substitution of the TATA-Box within the promoter region upstream of transcriptional start site 23 (TSS23) in the phage genome was performed to generate a non-infectious phage mutant (12). The resulting phage can still replicate and lyse the bacteria, but can no longer assemble intact phage particles. The promoter region upstream of TSS23 was amplified using primer pCG925gibfor/pCG925gibrev and cloned into the BamHI-digested pIMAY-Z vector in *E. coli* DC10B. The plasmid was used as a template for site-directed mutagenesis (SDM) with primer pCG910SDMfor/pCG910SDMrev according to the manufacturer’s instructions (Q5 Site-Directed Mutagenesis Kit, New England Biolabs). To confirm the substitution within the TATA box of the promoter, the plasmid pCG925 was sequenced and subsequently transformed into the desired *S. aureus* strains by electroporation. Genomic pIMAY-Z mutagenesis within the phage genome was performed as described before and final mutants confirmed by PCR and sequencing (39).

### Generation and propagation of phage lysates

Phage lysates were obtained by induction of single-lysogenic strains by the addition of subinhibitory MMC (300 ng mL^−1^) and subsequent sterile filtration (0.45 µm) of the supernatant. To propagate phages and achieve higher titers, phages were used to infect propagation strains at an MOI of 1 and incubated for 4 to 6 h until lysis of bacteria was visible. Phages were harvested by centrifugation, followed by sterile filtration (0.45 µm) of the supernatant. Phage titers were determined by plaque assays.

### Plaque assay

Phage titers were determined by the agar overlay method. Indicator strains were grown to an OD_600_ of 0.1, 250 µL bacterial culture was mixed with 10 mL phage top agar (Casamino acids 3 g L^−1^, yeast extract 3 g L^−1^, NaCl 5.9 g L^−1^, agar 7.5 g L^−1^) and poured onto NB_2_ agar plates supplemented with CaCl_2_. Serial dilution of sterile filtered (0.45 µm) phages in phage buffer (Tris 50 mM, CaCl_2_ 4 mM, MgSO_4_ 1 mM, NaCl 5.9 g L^−1^, gelatine 1 g L^−1^) was spotted onto a bacterial lawn and incubated overnight at 37°C. Phage titers were quantified as plaque-forming units per milliliter.

### Infection assay

Bacterial cultures of phage-free strains were grown to the exponential phase, 1 × 10^6^ bacteria were infected with phages at an MOI of 1 in a 24-well plate, and incubated at 37°C and 130 rpm. At time points 0, 1, 3, and 6 h, bacterial and phage numbers were determined. For bacterial quantification, colony-forming units (CFU) were enumerated by spotting serial dilutions onto TSA plates and overnight incubation at 37°C. Extracellular phage titers were quantified by qPCR on the circularized phage genome in sterile filtrated (0.45 µm) culture supernatants.

### Adsorption assay

Bacteria were grown to an OD_600_ of 1 to reach the exponential growth phase. For induction of AgrA, 100 nM AIP-I (autoinducing peptide I) was added to the culture (26). A total of 1 × 10^8^ bacteria were infected with 1 × 10^6^ phages in TSB supplemented with CaCl_2_ and incubated for 10 min at room temperature without shaking. Cultures were pelleted by centrifugation, and supernatants, containing unbound phages, were sterile filtrated and further analyzed by qPCR and plaque assay. For calculating the percentage of bound phages, a control condition without bacteria was included to determine phage titer after incubation (100% unbound phages).

### Prophage induction and replication

Phage induction and the resulting phage progeny replication were analyzed as described before (12). Briefly, single-lysogens were grown to the exponential phase and induced with a subinhibitory concentration of MMC (300 ng mL^−1^). To quantify spontaneous induction and following replication, cultures were incubated untreated. One hour post-induction, cell supernatants were harvested and sterile filtrated (0.45 µm), pellets were resuspended in TE buffer, and stored for further analysis by qPCR.

### Quantitative PCR for phage genome quantification

To quantify phage genomes within phage lysates, samples were treated with Proteinase K (AppliChem) for 1 h at 55°C, followed by inactivation for 10 min at 95°C. For quantification of phage genomes within the bacterial pellet, bacteria were lysed using a high-speed homogenizer (2 × 20 s, 6,500 rpm) and zirconia/silica beads (0.1 mm diameter). Lysed pellets were boiled for 10 min. Lysates and lysed pellets were diluted 1:10 in nuclease-free water for qPCR. qPCR was performed with QuantiFAST SYBR Green PCR Kit (Qiagen). For excised or extracellular phage genome quantification, primers spanning the reconstituted *attP* site were used. To quantify the excision of phage genomes from the bacterial genome, qPCR on reconstituted *hlb* was performed. Additionally, *recA* was quantified for normalization to the number of bacterial genomes for intracellular phage replication analysis.

Oligonucleotides used for qPCR are listed in Table S3.

### RNA isolation, RT-qPCR

For RNA isolation, bacteria were harvested and resuspended in 1 mL TRIzol (Thermo Fisher Scientific). Cells were lysed using a high-speed homogenizer (6,500 rpm) and zirconia/silica beads (0.1 mm diameter). RNA was isolated as recommended by the TRIzol manufacturer. A total of 5 µg of isolated RNA was DNase-treated (Roche) and subsequently diluted 1:10 in nuclease-free water for RT-qPCR.

To determine gene expression of target genes, RT-qPCR was performed with QuantiFast SYBR Green RT-PCR Kit (Qiagen) using the Quantstudio3 system (Applied Biosystems) with the recommended settings. Relative expression was calculated using the ∆∆CT method with *gyrB* as the housekeeping gene for normalization. Untreated wild-type bacteria were used as a control condition.

Oligonucleotides used for RT-qPCR are listed in Table S3.

### Promoter activity measurement

Bacteria from an overnight culture were inoculated to an initial OD_600_ of 0.05 and grown to an OD_600_ of 0.7. Subsequently, cultures were induced by the addition of subinhibitory MMC (300 ng mL^−1^). One hour post-induction, cells were adjusted to an OD_600_ of 2 in PBS, and 200 µL were transferred to a 96-well plate. Optical density (absorbance: 600 nm) and fluorescence (yellow, gpVenus: excitation 500 nm, emission 545 nm; blue, gpCerulean: excitation 434 nm, emission 485 nm) were measured using a Tecan Spark plate reader. Control bacteria without the plasmids were included for each strain to correct for strain-background fluorescence (strain blanked). Resulting values were normalized to OD_600_.

### Proteolysis assay

Skim milk agar plate assays were performed as described before (40). In brief, overnight cultures were grown, diluted to an OD_600_ of 1 in TSB, and 25 µL of bacterial suspension was filled into wells (5 mm diameter) cut in 2% skim milk agar plates. Proteolysis was quantified by measurement of clear areas surrounding the bacteria, after 48 h of incubation at 37°C.

### Influence of supernatant/proteases on phage titers

Cultures were grown to an OD_600_ of 0.5, and supernatants of 1 × 10^8^ bacteria were collected by centrifugation, followed by sterile filtration (0.45 µm). A total of 1 × 10^8^ phages were incubated in culture supernatants, titers of phage lysates were determined by plaque assay after 3 h of incubation at 37°C and 130 rpm. RN4220 and LS1 were used as indicator strains for Φ11 and Φ13, respectively. Phages were additionally incubated in fresh TSB as a control.

### Supercoiling assay

Assessment of the DNA supercoiling status of individual strains was performed by chloroquine gel electrophoresis essentially as described before (41). Strains were initially transformed by electroporation with the small staphylococcal plasmid pC194 and selected on chloramphenicol (10 µg mL^−1^). A total of 10 mL overnight cultures of respective transformants were used for plasmid purification using the GeneJet Plasmid Miniprep Kit (Thermo Fisher Scientific), including a 30 min preincubation step with lysostaphin (50 µg mL^−1^) in resuspension buffer at 37°C. Plasmid preparations were run on a 1% agarose (2xTBE) gel containing 2.5 µg mL^−1^ chloroquine (10 V, 16 h), which was subsequently washed twice with water for 30 min and stained with SYBR Safe for 30 min before visualization.

### Statistics

Statistical analyses were performed using GraphPad Prism 10.3.1 software. Information on the tests performed for each data set was added to figure captions; means ± SD are indicated in graphs. Differences with *P* < 0.05 were considered significant. All data represent values from independent biological replicates; statistical analysis was performed with at least three biological replicates (*n* = 3).

## SUPPLEMENTAL MATERIAL

Supplemental tables and figures jb.00279-25-s0001.docx Strain, plasmid information, and supplemental results.

ASM does not own the copyrights to Supplemental Material that may be linked to, or accessed through, an article. The authors have granted ASM a non-exclusive, world-wide license to publish the Supplemental Material files. Please contact the corresponding author directly for reuse.
