# Supplementary material for: Multiple effects of the bacterial DNA-binding protein SarA on the life cycle of Staphylococcus aureus phages
Source: J Bacteriol. 2025 Oct 16;207(11):e00279-25. doi: 10.1128/jb.00279-25 (PMC12632249; doi:10.1128/jb.00279-25)
Supplement: Supplemental tables and figures — Strain, plasmid information, and supplemental results. [file jb.00279-25-s0001.docx]

**Supplementary information**

**Table S1: Strains**

| Strain | Description | Reference/ Origin |
| --- | --- | --- |
| *Escherichia coli* |  |  |
| DC10B |  | (1) |
| *Staphylococcus aureus* |  |  |
| 8325-4 (RN0450) | NCTC8325 cured of Φ11, Φ12 and Φ13 | (2) |
| 8325-4 Φ13K | Single-lysogen, *kan^R^* | (3) |
| SH1000 | *rsbU* repaired derivative of 8325-4 | (4)  Susanne Engelmann, TU Braunschweig, Germany |
| RN6390 *sarA* (ALC1342) | *sarA*::*ermC, sarA* nt 586 to 1107 replaced by *ermC* gene, | (5) |
| SH1000 *sarA* | *sarA::ermC*, via transduction | This study |
| SH1000 *sarA* p*sarA* | *sarA::ermC*, complemented with integrative vector carrying *sarA* locus (p*sarA*) | This study |
| SH1000 Φ13K | Single-lysogen, *kan^R^* | (3) |
| SH1000 Φ13K *sarA* | *sarA::ermC, s*ingle-lysogen, *kan^R^* | This study |
| SH1000 Φ13K *sarA* p*sarA* | *sarA::ermC*, single-lysogen, complemented with p*sarA*, *kan^R^* | This study |
| SH1000 Φ13K-*rep* | Single-lysogen, carrying replication deficient phage mutant (3), *kan^R^* | This study |
| SH1000 Φ13K-*rep sarA* | *sarA::ermC,*  single-lysogen, carrying replication deficient phage mutant, *kan^R^* | This study |
| SH1000 Φ11E | Single-lysogen, *erm^R^* | This study |
| SH1000 Φ11E *sarA* | *sarA::kan, erm^R^* | This study |
| Newman-c | Phage-cured | (6) |
| Newman-c *sarA* | *sarA::ermC* | This study |
| Newman-c *sarA* p*sarA* | *sarA::ermC*, complemented with integrative vector carrying *sarA* locus (p*sarA*) | This study |
| RN6911 | *agr::tetM* | (7) |
| SH1000 *agr* | *agr::tetM*, via transduction | This study |
| SH1000 Φ13K *agr* | *agr::tetM, kan^R^* | This study |
| SH1000 Φ13K-*TSS23* | Non-infectious phage mutant | This study |
| SH1000 Φ13K-*TSS23* *sarA* | Non-infectious phage mutant, *sarA::ermC* | This study |
| 8325-4 Φ11E | Single-lysogen, *erm^R^* | (8)  Hanne Ingmer, Copenhagen, Denmark |
| LS1 |  | (9)  Löffler, Münster, Germany |
| RN4220 | restriction deficient derivate of 8325-4, rK-mK+ | (10) |
| CYL316 | RN4220 (pYL112Δ19), L54 int gene | (11) |

**Table S2: Phages**

| Phage Lysates | Resistance casette | Propagation/ Indicator strain | Reference |
| --- | --- | --- | --- |
| Φ13K | *kan^R^* | LS1 |  |
| Φ13K-*int* | *kan^R^* | LS1 | (12) |
| Φ11 |  | RN4220 |  |
| Φ11E | *erm^R^* | RN4220 | (8) |

**Table S3: Oligonucleotides**

| Oligonucleotide | Sequence | Used for |
| --- | --- | --- |
| SarA DIGfor | CAATGATTGCTTTGAGTTGT | Control PCR *sarA* |
| SarA DIGrev | CGTTTATTTACTCGACTCAA | Control PCR *sarA* |
| Sa5intfor | AAAGATGCCAAACTAGCTG | Control PCR Φ11 |
| Sa5intrev | CTTGTGGTTTTGTTCTGG | Control PCR Φ11 |
| pCG921gibfor | tcgagctcggtacccgggTAACTTTT  AGCTTATCATTTTAACTTGT | Cloning pCG921 |
| pCG921gibrev | tgcaggtcgactctagagTATGTGA  TATATAAACCTAGGGCA | Cloning pCG921 |
| pLL39newfor | GTAATGGGCCCAATCACTAGTG | Control PCR pCG921 |
| pLL39rev2016 | ACGCCAGAAGATACAAAGCA | Control PCR pCG921 |
| pCG921insidecontrolfor | GGGCAAATGTATCGAGCAAGA | Control PCR pCG921 |
| Scv2.1 | TGTGCCATGATAACAGCACG | Control PCR pCG921 |
| Scv4 | ACCCAGTTTGTAATTCCAGGAG | Control PCR pCG921 |
| Scv8 | GCACATAATTGCTCACAGCCA | Control PCR pCG921 |
| Scv1 | GCAACACCACATAATGGTTCAC | Control PCR pCG921 |
| pcIyfpgibfor | GCTGGCGGCCGCTGCATGCAAG  TTCACGTATCATGAACT | Cloning pCG748 |
| pcIyfpgibrev | CATAAATAATCATCCTCCTAAGGT  TTGATAACTTCATAATAAAGCTTGT | Cloning pCG748 |
| pCG789gibfor | gagctggcggccgctgcatgTTTTGTATTGAC  TTGATTCAAAACAAGGT | Cloning pCG789 |
| pCG789gibrev | cataaataatcatcctcctaagCAAGTTCACG  TATCATGAACT | Cloning pCG789 |
| pumuCYFPgibfor | CGGCCGCTGCATGGCATGCCCAA  ACCTCCTAATCATTA | Cloning pCG762 |
| pumuCYFPgibrev | AAATAATCATCCTCCTAAGGTCGA  CTACTTGAATCTTATT | Cloning pCG762 |
| pcIyfpcontrolrev | TGACAAGTGTTGGCCATGGA | Control PCR |
| pcIyfpoutsidecontrolfor | GGACAGGTATCCGGTAAGCG | Control PCR |
| pcapmutseqfor | TTTGTGATGCTCGTCAGGGG | Control PCR |
| pCG925gibfor | aattcctgcagcccggggCTATGACTATT  GTATTTGCTATATTGCT | Cloning pCG925 |
| pCG925gibrev | gccgctctagaactagtgGCACATCACTC  CTTGTCGAC | Cloning pCG925 |
| pCG910SDMfor | cggccGCTGTGTAGCAAAACATTTA  TATTTC | Cloning pCG925 |
| pCG910SDMrev | cggcgAAAAACAATATGTAGCATCA  AAATTAG | Cloning pCG925 |
| pCG925outsidecontrolfor | GCGGAGGTAAGTGAGTGA | Cloning pCG925 |
| pCG925outsidecontrolrev | GGATGACCACATCGCTTCA | Cloning pCG925 |
| pCG849i1gibfor | tcgataagcttgatatcgTAAACAAAAAA  CGCCTACAAGTGT | Cloning pCG849 |
| pCG849i1gibrev | TTAACTTTATACAAAATTGGCAAA  AAATAATAAGGGT | Cloning pCG849 |
| pCG849i2gibfor | TGCCAATTTTGTATAAAGTTAATT  ATAAAGCCGGAAAACCT | Cloning pCG849 |
| pCG849i2gibrev | GATCCCCCGGGCTGCAGGGTTC  TTACCATTTTTCTCTATTTTTGT | Cloning pCG849 |
| pIMAYcontrolfor | CCAGCCCCCTCACTACAT | Control PCR pCG849 |
| pIMAYcontrolrev | ATCACCCGACGCACTTTG | Control PCR pCG849 |
| pCG849outsidectlfor | CAACTGGTGCTGGCATAGGA | Control PCR *int* |
| pCG849outsidectlrev | TCGATCATGTCCAGCACCAC | Control PCR *int* |
| pCG844insidecontrolfor | TTTTGGCTTGTACCGTTCAC | Control PCR *int* |
| circlefor | TTTTATTTTATATGGGGTATTATTGA | qPCR (Φ13 *attP*) |
| circlerev | GTGTATTCTCATTTGTTAGAAGAAAA | qPCR (Φ13 *attP*) |
| Sa5intStafor | ACAAACGAAAAATGAAGCGT | qPCR (Φ11) |
| Sa5intStarev | AGTCTAGTTAGCTGACGAGA | qPCR (Φ11) |
| recAF1 | GCTCAAGCATTAGGCGTAGAT | qPCR (*recA*) |
| recA661 | ATTTTAATGCACGTCCACCTGG | qPCR (*recA*) |
| hlb258 | ATTAGTTGGTGCACTTACTG | qPCR (*hlb*) |
| hlb675 | GCTATCATTATCGAATCCAC | qPCR (*hlb*) |
| tarMqPCRfor | CAAGGTAAAATGGATCGAAGAAC | RT-qPCR (*tarM*) |
| tarMqPCRrev | GTAGGCAATATATGTACCAGTC | RT-qPCR (*tarM*) |
| tarSqPCRfor | TAGTGCGTATGTTTCACCTG | RT-qPCR (*tarS*) |
| tarSqPCRrev | AAGTCTCCTAGAGCATTAATCC | RT-qPCR (*tarS*) |
| umuCqPCRfor | TCTAAGATTGCATTGCGTTA | RT-qPCR (*umuC*) |
| umuCqPCRrev | CATATTAGAACCAATGCCCA | RT-qPCR (*umuC*) |
| SAOUHSC02196for2 | CACGAATCAAAACGGCATTA | RT-qPCR (*terminase*) |
| SAOHHSC02196rev2 | ACAACAATCGAATCAATGGC | RT-qPCR (*terminase*) |
| SAOUHSC_02200qPCRfor | GGCACGACTAGCAATAAA | RT-qPCR (*ltr*) |
| SAOUHSC_02200qPCRrev | GTCTCTGCCTATATCAAGAAT | RT-qPCR (*ltr*) |
| SAOUHSC_02191for | TTTGCATCTTCGATTGCTTC | RT-qPCR (*mcp*) |
| SAOUHSC_02191DIGrev | TACGACAATCAGAAGTTGCA | RT-qPCR (*mcp*) |
| Gyr574 | AGTCTTGTGACAATGCGTTTACA | RT-qPCR (*gyr*) |
| Gyr297 | TTAGTGTGGGAAATTGTCGATAAT | RT-qPCR (*gyr*) |
| SAOUHSC02234DIGfor | TAATACGACTCACTATAGGGAGAT  GCAAAATTGTACTGAGTGC | RT-qPCR (*mor*) |
| SAOUHSC02234DIGrev | ATGTGTTACGACTACTCACG | RT-qPCR (*mor*) |
| cIqPCRfor | AGAACGTCAAGATGAAACGA | RT-qPCR (*cI*) |
| cIqPCRrev | AATTCTTCTCCTATGCCAGC | RT-qPCR (*cI*) |

**Table S4: Plasmids**

| Plasmid | Description | Resistance casette | Reference/ Origin |
| --- | --- | --- | --- |
| pLL39 | Integrative vector, integrates into *geh* | *tet* | (13) |
| pIMAY-Z | Mutagenesis vector | *cm* | (14) |
| pIMAY | Mutagenesis vector | *cm* | (1) |
| pCG921 | Complementation vector p*sarA*, pLL39 containing *sarA* locus | *tet* | This study |
| pCG725 | P*_cap_*-*yfp* | *cm* | (15) |
| pCG733 | P*_cap_*-*cfp* | *cm* | (15) |
| pCG762 | Promoter construct P*_umuC_*-*yfp* | *cm* | This study |
| pCG748 | Promoter construct P*_cI_*-*yfp* | *cm* | This study |
| pCG789 | Promoter construct P*_mor_*-*yfp* | *cm* | This study |
| pCG925 | Mutagenesis vector for p23 TATA Box mutation (pIMAY-Z), non-infectious phage mutant | *cm* | This study |
| pCG849 | Mutagenesis vector for *int* deletion (pIMAY) | *cm* | This study |
| pCG32 | *Int* complementation | *cm* | (16) |

**
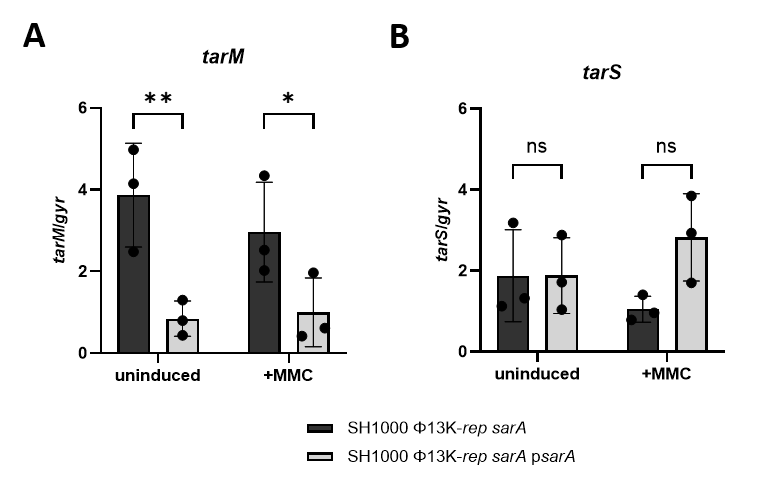
**

**Figure S1: *tarM* and *tarS* expression in *sarA*-complemented background.** Gene expression analysis of *tarM* (α-1,4 GlcNAc) and *tarS* (β-1,4 GlcNAc) under uninduced and induced (+MMC) conditions. Single-lysogenic SH1000 *sarA* (*sarA*::*ermC* deletion) and SH1000 *sarA* p*sarA* (*sarA* complementation), containing the replication-deficient Φ13K-*rep* mutant, were grown to exponential growth phase, followed by prophage induction with subinhibitory mitomycin C (MMC), and incubation for 60 min. RNA was isolated, *tarM* and *tarS* transcripts were quantified by qRT-PCR and normalized to *gyr* expression.

**Figure S2: Φ13K phage replication is not *agr* dependent.** Phage replication in SH1000 Φ13K and SH1000 Φ13K *agr* (*agr*::*tetM* deletion) under uninduced and induced (+MMC) conditions. Single-lysogenic strains were induced with subinhibitory mitomycin C (MMC) in exponential growth phase and incubated for further 60 min. Phage lysates were harvested by centrifugation and following sterile filtration of the supernatant. Phage numbers were determined by qPCR on the circularization site of the phage genome (*attP*) and presented per ml. Data shown are mean ± SD (n = 3). Statistical analysis was determined by 2way ANOVA test (ns > 0.05).

**
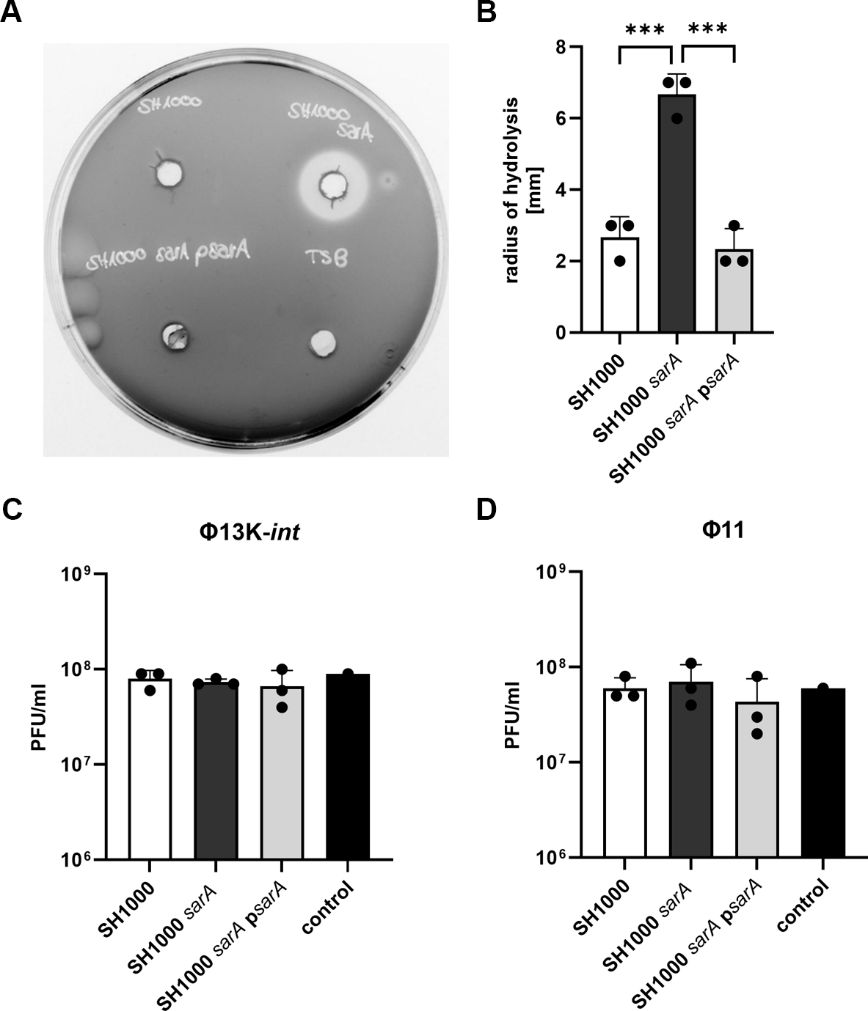
**

**Figure S3: Protease-dependent phage decay is not *sarA* dependent.** (A,B) Proteolysis activity of SH1000 (wild type), SH1000 *sarA* (*sarA*::*ermC* deletion) and SH1000 *sarA* p*sarA* (*sarA* complemented). Overnight cultures were diluted to OD_600_ of 1 and 25 µl of bacterial suspensions were filled into wells cut into skim milk agar plates. (A) Pictures were taken after 24 h of incubation at 37°C showing proteolysis haloes. (B) Proteolysis radii were measured after 48 h of incubation at 37°C. Data shown are mean ± SD (n = 3). Statistical significance was determined by ordinary one-way ANOVA. (C,D) Phage numbers after incubation in spent media of SH1000, SH1000 *sarA* and SH1000 *sarA* p*sarA* 1x10^8^ phages of (C) Φ13K-*int* or (D) Φ11 were incubated for 3 h in the supernatant of 1x10^8^ bacteria, grown to OD_600_ = 0.5. Titers of phage lysates were determined by plaque assay on bacterial lawn of LS1 (Φ13K-*int*) or RN4220 (Φ11) as indicator strains. Phages incubated in fresh TSB were included as control. Data shown are mean ± SD (n = 3). Statistical significance was determined by ordinary one-way ANOVA (ns > 0.05).


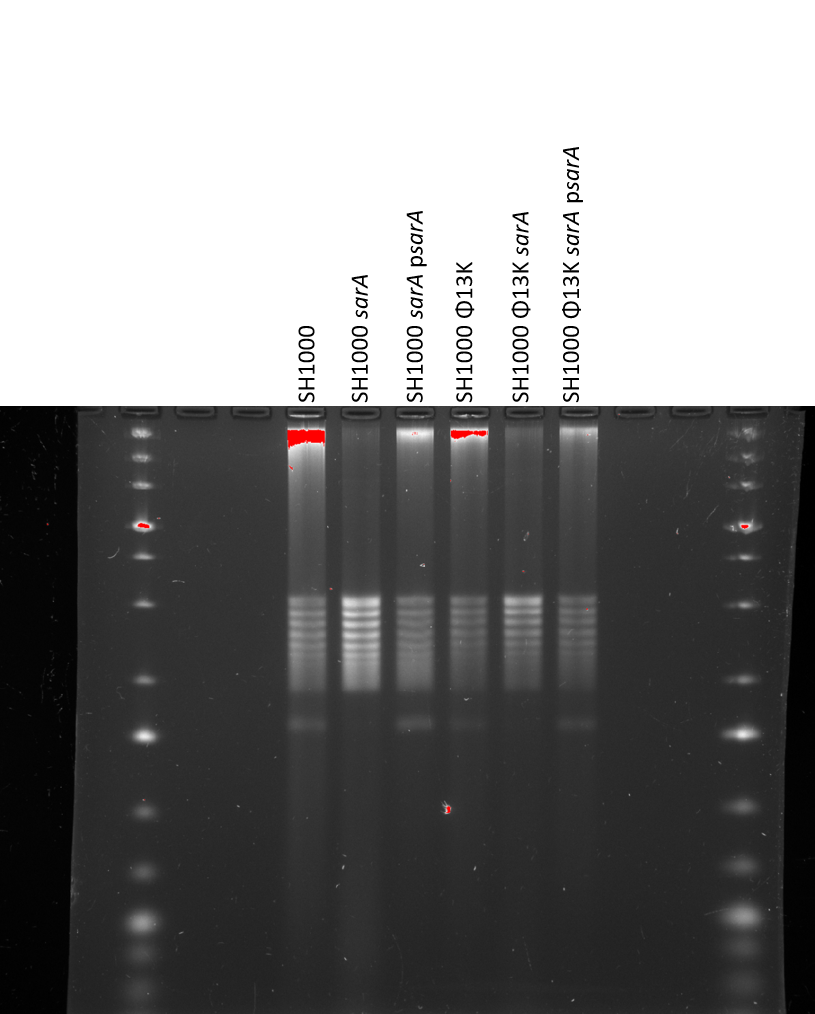


**Figure S4: No apparent effect of *sarA* on DNA supercoiling.** Plasmid (pC194) preparations from SH1000, *sarA* derivatives, and Φ13K lysogens were separated by chloroquine agarose gel electrophoresis. GeneRuler 1 kb Plus DNA Ladder (Thermo Scientific) was used in first and last lane.

**Figure S5: Higher SOS-response activation in *sarA* deleted background, determined by *umuC* gene expression.** Gene expression analysis of *umuC* under uninduced and induced (+MMC) conditions. Single-lysogenic SH1000 and SH1000 *sarA* (*sarA*::*ermC* deletion), containing replication deficient phage mutant (*rep*), were grown to exponential growth phase, induced with subinhibitory mitomycin C (MMC) and incubated for 60 min. RNA was isolated, *umuC* transcripts were measured by qRT-PCR and normalized to *gyr*. Data shown are mean ± SD (n = 3). Statistical analysis was determined by 2way ANOVA test (*p-value < 0.05, ns > 0.05).

**References**

1. Monk IR, Shah IM, Xu M, Tan MW, Foster TJ. 2012. Transforming the untransformable: application of direct transformation to manipulate genetically *Staphylococcus aureus* and *Staphylococcus epidermidis*. mBio 3:e00277-11.

2. Novick R. 1967. Properties of a cryptic high-frequency transducing phage in *Staphylococcus aureus*. Virology 33:155-66.

3. Rohmer C, Dobritz R, Tuncbilek-Dere D, Lehmann E, Gerlach D, George SE, Bae T, Nieselt K, Wolz C. 2022. Influence of *Staphylococcus aureus* Strain Background on Sa3int Phage Life Cycle Switches. Viruses 14:2471.

4. Horsburgh MJ, Aish JL, White IJ, Shaw L, Lithgow JK, Foster SJ. 2002. sigmaB modulates virulence determinant expression and stress resistance: characterization of a functional rsbU strain derived from *Staphylococcus aureus* 8325-4. J Bacteriol 184:5457-67.

5. Cheung AL, Schmidt K, Bateman B, Manna AC. 2001. SarS, a SarA homolog repressible by agr, is an activator of protein A synthesis in *Staphylococcus aureus*. Infect Immun 69:2448-55.

6. Bae T, Baba T, Hiramatsu K, Schneewind O. 2006. Prophages of *Staphylococcus aureus* Newman and their contribution to virulence. Mol Microbiol 62:1035-47.

7. Novick RP, Ross HF, Projan SJ, Kornblum J, Kreiswirth B, Moghazeh S. 1993. Synthesis of staphylococcal virulence factors is controlled by a regulatory RNA molecule. Embo j 12:3967-75.

8. Quiles-Puchalt N, Martínez-Rubio R, Ram G, Lasa I, Penadés JR. 2014. Unravelling bacteriophage ϕ11 requirements for packaging and transfer of mobile genetic elements in *Staphylococcus aureus*. Mol Microbiol 91:423-37.

9. Bremell T, Abdelnour A, Tarkowski A. 1992. Histopathological and serological progression of experimental *Staphylococcus aureus* arthritis. Infect Immun 60:2976-85.

10. Kreiswirth BN, Lofdahl S, Betley MJ, O'Reilly M, Schlievert PM, Bergdoll MS, Novick RP. 1983. The toxic shock syndrome exotoxin structural gene is not detectably transmitted by a prophage. Nature 305:709-12.

11. Lee CY, Buranen SL, Ye ZH. 1991. Construction of single-copy integration vectors for *Staphylococcus aureus*. Gene 103:101-5.

12. Lehmann E, van Dalen R, Gritsch L, Slavetinsky C, Korn N, Rohmer C, Krause D, Peschel A, Weidenmaier C, Wolz C. 2024. The Capsular Polysaccharide Obstructs Wall Teichoic Acid Functions in *Staphylococcus aureus*. J Infect Dis 230:1253-1261.

13. Luong TT, Lee CY. 2007. Improved single-copy integration vectors for *Staphylococcus aureus*. J Microbiol Methods 70:186-90.

14. Monk IR, Tree JJ, Howden BP, Stinear TP, Foster TJ. 2015. Complete Bypass of Restriction Systems for Major *Staphylococcus aureus* Lineages. mBio 6:e00308-15.

15. Keinhörster D, Salzer A, Duque-Jaramillo A, George SE, Marincola G, Lee JC, Weidenmaier C, Wolz C. 2019. Revisiting the regulation of the capsular polysaccharide biosynthesis gene cluster in *Staphylococcus aureus*. Mol Microbiol 112:1083-1099.

16. Mainiero M, Goerke C, Geiger T, Gonser C, Herbert S, Wolz C. 2010. Differential target gene activation by the *Staphylococcus aureus* two-component system saeRS. J Bacteriol 192:613-23.
